# A Review of Emerging Technologies in Ultra-Smooth Surface Processing for Optical Components

**DOI:** 10.3390/mi15020178

**Published:** 2024-01-25

**Authors:** Wei Li, Qiang Xin, Bin Fan, Qiang Chen, Yonghong Deng

**Affiliations:** 1 National Key Laboratory of Optical Field Manipulation Science and Technology, Chinese Academy of Sciences, Chengdu 610209, China; liwei_ioe@163.com (W.L.); chq@ioe.ac.cn (Q.C.); dengyh@std.uestc.edu.cn (Y.D.); 2 Institute of Optics and Electronics, Chinese Academy of Sciences, Chengdu 610209, China; 3 University of Chinese Academy of Sciences, Beijing 100049, China; 4 School of Optoelectronic Science and Engineering, University of Electronic Science and Technology of China, Chengdu 610054, China

**Keywords:** ultra-smooth surface processing, ultra-precision optical components, surface roughness, material removal mechanism

## Abstract

Advancements in astronomical telescopes and cutting-edge technologies, including deep ultraviolet (DUV) and extreme ultraviolet (EUV) lithography, have escalated demands and imposed stringent surface quality requirements on optical system components. Achieving near-ideal optical components requires ultra-smooth surfaces with sub-nanometer roughness, no sub-surface damage, minimal surface defects, low residual stresses, and intact lattice integrity. This necessity has driven the rapid development and diversification of ultra-smooth surface fabrication technologies. This paper summarizes recent advances in ultra-smooth surface processing technologies, categorized by their material removal mechanisms. A subsequent comparative analysis evaluates the roughness and polishing characteristics of ultra-smooth surfaces processed on various materials, including fused silica, monocrystalline silicon, silicon carbide, and sapphire. To maximize each process’s advantages and achieve higher-quality surfaces, the paper discusses tailored processing methods and iterations for different materials. Finally, the paper anticipates future development trends in response to current challenges in ultra-smooth surface processing technology, providing a systematic reference for the study of the production of large-sized freeform surfaces.

## 1. Introduction

The recent rapid development of advanced optoelectronic systems, such as large astronomical telescopes and DUV and EUV lithography machines, has necessitated urgent enhancements in the performance of the lithography machine objective lens, telescope refraction rate, and beam control system. These systems impose increasingly stringent performance requirements. Consequently, the optical components of the system must exhibit sub-nanometer roughness and be free from subsurface damage. Stringent manufacturing specifications include ensuring lattice integrity and minimizing surface flaws and residual stress [1,2]. In the case of EUV lithography, the 13.5 nm wavelength light is strongly absorbed by the vast majority of optical materials. Therefore, it is imperative to enhance the surface quality of the optical elements in the beam control system to ensure that the light approaches the ideal state after refraction and reflection. This improvement aims to enhance the resolution, etching accuracy, and product yield in the lithography machine. Similarly, in the manufacture of astronomical telescopes, the polishing of large-sized optical components with small tools introduces surface errors that can significantly degrade the imaging resolution of the telescope system [3]. Thus, reducing the surface shape inaccuracy of optical components and achieving sub-nanoscale surface roughness are essential in increasing the system’s resolution [4]. Similar to this, telescopes utilized for laser emission and reception between two satellites separated by millions of kilometers have recently garnered significant attention in the field of gravitational wave detection engineering. This heightened interest stems from the direct impact of the wavefront quality and stability on the accuracy of gravitational wave signal calculations, subsequently influencing the strength and phase distribution of the transmitted and received signals [5,6]. Gyroscopes serve as integral components in spacecraft attitude control within inertial navigation systems (INS). The quality factor can experience an increase of approximately two orders of magnitude when the root mean square value of surface roughness is reduced by one order of magnitude [7,8]. In order to enhance their performance, there is a pressing need for optical components featuring extremely smooth optical surfaces. Additionally, surface irregularities such as pitting, scratches, or bubbles can lead to energy loss and beam scattering. These irregularities can even cause optical components to absorb beam energy, undergo thermal deformation, and ultimately cease functioning. The improvement of the damage threshold in optical components through ultra-smooth surface processing plays a crucial role in advancing inertial confinement fusion engineering—for instance, the United States’ National Ignition Facility (NIF) [9,10].

Conventional rigid contact polishing involves the utilization of polishing instruments affixed with abrasive particles, such as grinding wheels, disks, and abrasive belts, to refine the workpiece surface [11]. In an investigation exploring the influence of the organizational structure of a diamond grinding wheel on its performance, Y. Ding et al. observed that the porosity of a grinding wheel could enhance its folding strength but concurrently accelerate wear on the wheel itself [12]. Furthermore, a grinding wheel with higher porosity was found to dislodge particles and markedly diminish the surface quality. Q. Y. Liang et al. employed a diamond sand belt for the grinding of titanium alloy blades and identified that the wear of the sand belt primarily resulted from the loss or adherence of abrasive particles [13]. Researchers have proposed a grinding wheel with a circular arc transition at the edge to mitigate linear defects arising from the grinding wheel polishing of fused silica. The corresponding dressing method was subsequently employed to control the depth of sub-surface defects within 2.5 μm, leading to a notable enhancement in surface quality and minimizing defects in the grinding of fused silica components [14].

Residual stress, surface defects, and sub-surface damage are prevalent issues associated with traditional rigid contact polishing, and the stability of material removal is significantly affected by polishing tool wear [15]. To attain ultra-smooth surfaces, researchers have increasingly directed their attention towards flexible contact polishing and non-contact polishing. Techniques such as chemical mechanical polishing, elastic emission polishing, magnetorheological polishing, ion beam polishing, and plasma polishing are considered effective in mitigating these challenges. Additionally, novel composite processing technologies, rooted in these techniques, have rapidly emerged [16]. This paper systematically presents and analyzes ultra-smooth surface processing technology. It begins by introducing the fundamental material removal mechanism of ultra-smooth surface processing. Building upon this foundation, it delves into the principles and advancements of chemical mechanical polishing, elastic emission polishing, ion beam polishing, plasma polishing, and magnetorheological polishing technologies in recent years. Furthermore, it compares and analyzes the ultra-smooth surface quality achieved by different methods on various materials, summarizing the strengths and limitations of each polishing approach. Finally, the paper concludes with an overview of ultra-smooth surface processing technology and offers insights into its prospective future development.

## 2. Ultra-Smooth Surface Processing Technology

Advancements in processing technology have surpassed the conventional 1 nm surface roughness, which is now deemed insufficient to meet the production requirements of precision optical systems. For instance, the optical glass surface roughness utilized in light reflection and neutron-guiding optical devices in nuclear physics must be less than 0.8 nm to ensure that the radiation wavelength is lower than the visible light wavelength [17]. Integrated circuit production employs less than 0.5 nm surface roughness on SiC substrates to minimize electron energy loss at the interface and enhance device performance in compliance with application requirements [18]. Achieving surface roughness better than 0.5 nm on optical element surfaces is crucial for DUV lithography objectives to reduce light surface scattering, increase luminous flux, and enhance reflectivity [19]. The EUV reflective projection lithography system demands accuracy of 0.1 nm for individual components [20]. Consequently, the definition of ultra-smooth surfaces now includes characteristics such as surface roughness less than 0.5 nm, high surface shape accuracy, minimal surface defects and sub-surface damage, lattice integrity, and low surface residual stress [21]. The process capable of achieving ultra-smooth surfaces is termed ultra-smooth surface processing technology, driven by the escalating demand for such surfaces in optical systems. Owing to the wide range of optical component materials, this article primarily reviews the processing methods and advancements of the ultra-smooth surface processing procedures of fused quartz, monocrystalline silicon, silicon carbide, and sapphire.

In-depth exploration into the formation mechanism of ultra-smooth surfaces has led to the emergence of various processing methods that systematically incorporate principles from fluid mechanics, electromagnetics, chemistry, and energy beam processing to activate surface atoms. Applying the precision grinding principle to ultra-smooth surface machining necessitates ultra-precise motion machines for the processing of atoms on the material surface, coupled with exceptionally fine and wear-resistant cutting tools to achieve atomic-level material removal. However, meeting these requirements remains challenging with current technology. Presently, chemical reaction and energy beam action are the two primary techniques to achieve atomic-level elimination. Chemical reaction, operating under hydrodynamic pressure and mechanical force, utilizes substances in the polishing pad or polishing liquid to react with surface atoms, forming chemical bonds with higher binding energy. It then selectively disconnects chemical bonds with lower binding energy between surface atoms. The principal components of chemical reaction include elastic emission polishing and chemical mechanical polishing. On the other hand, the energy beam effect involves directing energy beams or highly active particles onto surface atoms, initiating reactions that lead to the breaking of surface atom bonds and subsequent migration. This process achieves atomic-level removal through sputtering or the generation of volatile products, resulting in a reduction in surface roughness to sub-nanometer levels [22]. Key applications of the energy beam effect encompass ion beam polishing and plasma polishing. Moreover, alternative methods for material removal include the utilization of flexible contact wear and fluid action. Atomic cluster removal, exemplified in processes like magnetorheological polishing, can nevertheless achieve a reduction in surface roughness to sub-nanometer levels under specific process conditions, even though the focus is not on individual atoms. In recent years, these processing methods have played a pivotal role in achieving atomic-level material removal processes, successfully yielding ultra-smooth surfaces.

## 3. Atomic-Level Removal of Materials under Chemical Reaction

**Figure 1:**
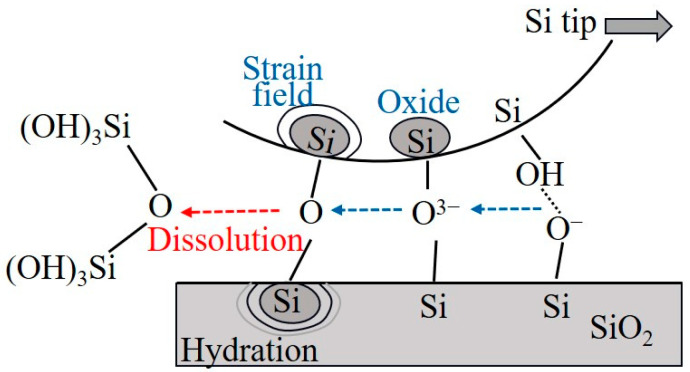
Surface chemistry bond removal model [26].

A pivotal stage in the ultra-smooth surface machining process involves the chemical removal of atomic surface material. To regulate the chemical reactions occurring during polishing and enhance the processing quality, active ingredients (oxidants, abrasive particles, additives, etc.) capable of chemically reacting with the workpiece’s surface are introduced into the polishing fluid or polishing pad. Researchers have extensively investigated the mechanism of chemical reaction in the processing of ultra-smooth surfaces of optical components. The currently acknowledged theory for the chemical removal of surface materials is Cook’s chemical bond removal model [23]. This model delineates the chemical activity into two steps: the active component forms a bond with the hydroxylated surface atoms after the optical element’s reaction with water produces hydroxylation. Subsequently, mechanical force disrupts the chemical link between the surface atoms with lower binding energy, enabling material removal at the atomic level. J. Yu et al. demonstrated that water molecules were involved in the removal of monocrystalline silicon atomic materials, but the precise reaction process was not conclusive, by analyzing the chemical composition of the wear debris, wear zone, and non-wear zone of monocrystalline silicon materials using time-of-flight secondary ion mass spectrometry (ToF-SMS) [24]. In a study using an atomic force microscope, F. Katsuki et al. scraped the surface of silicon dioxide with a monocrystalline silicon probe. The experimental results indicated that the removal volume of microscopic materials from the monocrystalline silicon probe and silicon dioxide was nearly equal in molar number, suggesting a chemical interaction process during contact and sliding [25,26]. As shown in Figure 1, silicic acid molecules dissolved in the solution were ultimately formed by the silicon atoms on the monocrystalline silicon probe’s surface. Additionally, X. Shi et al. mechanically and chemically polished fused silica using SO_2_ polishing liquid to comprehend the bonding process between the abrasive particle’s surface atoms and the workpiece’s surface atoms, along with the removal process under the influence of mechanical force. Surface characterization using infrared spectroscopy both before and after polishing revealed clear spectral peaks in the corresponding Si-O, Si-OH, and Si-O-Si regions on the polished surface of the acid SO_2_ slurry. This finding supports the atomic removal model via the formation of Si-O-Si bonds, indicating the occurrence of chemical reactions during the processing process [27]. Clearly, as the understanding of the mechanism underlying chemical material removal advances, the chemical bond removal model has been systematically established and verified step by step.

### 3.1. Chemical Mechanical Polishing

**Figure 2:**
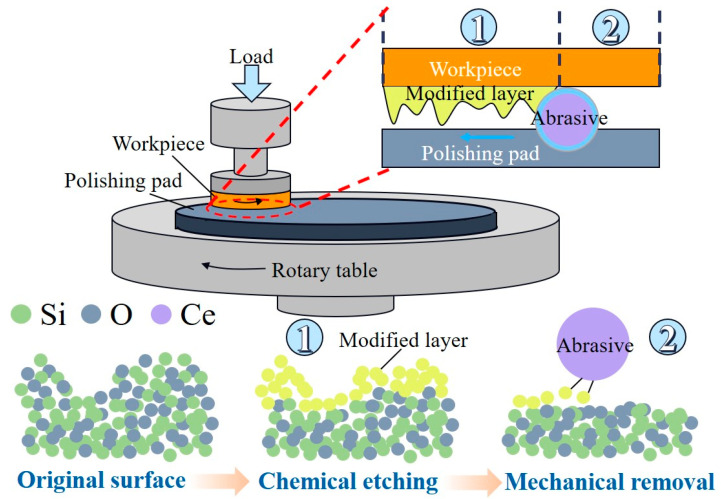
Illustration of chemical mechanical polishing [28,29].

Chemical mechanical polishing (CMP) is a method employed to generate ultra-smooth surfaces by inducing a reaction between the surface atoms of the workpiece and the active ingredients (such as oxidants, abrasives, additives, and pH regulators) present in a polishing solution or pad. Subsequently, mechanical action is integrated with the chemical reaction to yield a surface with ultra-low roughness. As depicted in Figure 2, CMP comprises essential components including the polishing solution, polishing disc, and additional control apparatus.

**Figure 3:**
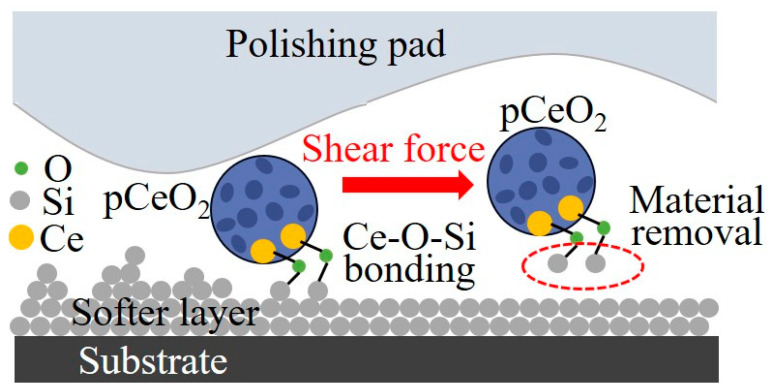
Material removal mechanism of pCeO_2_ nanospheres [36].

Robert originally introduced CMP for the polishing of semiconductor materials in 1965. His work underwent further exploration by IBM and other researchers [30]. In recent years, the research focus on polishing fluids has steadily advanced to achieve materials with extreme smoothness. Typical ingredients in polishing fluid encompass abrasive particles, pH adjusters, chemical additives, and deionized water [31]. The shape, structure, and physicochemical characteristics of abrasive particles significantly influence the surface roughness and MRR. Silicon dioxide (SiO_2_) and cerium oxide (CeO_2_) are frequently used abrasives. A SiO_2_ abrasive, with a more regular spherical form and size [32], can yield an ultra-smooth surface with roughness of less than 0.2 nm on SiC materials [33]. However, its industrial applicability is limited due to the MRR of approximately 1.0 µm/h. CeO_2_, with its irregular shape, wide particle size distribution, and tendency to aggregate, has the potential for a higher MRR than SiO_2_ but is more susceptible to surface damage or defects [34,35]. Efforts to enhance the CMP processing quality include doping additional components into abrasives to alter their morphology. Porous CeO_2_ nanospheres doped with Zr and Gd, for example, were created by A. Chen et al., resulting in a reasonably constant structure and a high specific area. The use of CeZrO_2_ as a new abrasive for CMP decreased the surface roughness of monocrystal silicon to Ra 0.11 nm [36], as seen in Figure 3. However, its average MRR was only 88 nm/min, limiting its processing efficiency. Researchers have explored the impact of CeO_2_ abrasive doping with Y, La, and Pr elements on the polishing morphology. As the doping amount increased, the abrasive’s morphology changed from spherical to octahedral, and the particle size dropped. An increased Ce^3+^ concentration and modified abrasive morphology were identified as the primary factors contributing to the improved polishing efficiency after doping [37]. The MRR of a CeO_2_ abrasive doped with Y reached a maximum of 630 nm/min. Fused silica achieved surface roughness of Ra 0.359 nm with a maximum MRR of 630 nm/min. Similarly, N. Xu et al. created CeO_2_ abrasives with a porous octahedron morphology, achieving surface roughness of Ra 0.396 nm using a CeO_2_-Ar/H_2_ (H_2_ concentration of 5%) abrasive on fused silica, with an improved MRR of 691 nm/min [38].

While doping can enhance material removal rates by altering the abrasive particle shape and increasing Ce^3+^, it negatively impacts the polishing efficiency as the abrasive particle size decreases. The MRR exhibits a negative trend with an increasing doping amount, even gradually declining. Consequently, the selection of the appropriate abrasive particle shape and size is crucial, depending on the desired outcome. Reducing the grinding particle size further improves the surface quality to achieve ultra-low surface roughness. Z. Wu et al. processed fused silica, producing an ultra-smooth surface with surface roughness of 0.086 nm, using ultra-fine CeO_2_ with low agglomeration strength, a high Ce^3+^ concentration, and a particle size smaller than 4 nm. CeO_2_ participated in CMP as a primary particle, as observed by comparing the length variations of CeO_2_’s secondary particles before and after CMP [39]. For the first time, the origin of submicron flaws on the surface of fused silica was investigated based on the agglomeration strength of secondary CeO_2_ particles, providing a foundation for the prediction of the quantity of surface defects. Z. Tan et al. used various physical and chemical techniques to further reduce the size of polishing particles, improving the polishing liquid performance and consistently producing an ultra-smooth surface with roughness of 0.4 Å on fused silica [40]. Importantly, the experiment demonstrated that the densification process resulting from the longitudinal pressure in the polishing process enhanced the surface uniformity and promoted the creation of ultra-smooth surfaces. This finding contradicted the conventional understanding, which held that the longitudinal pressure in the traditional polishing process should be kept to a minimum, and it has significant implications for other ultra-smooth surface processing technologies.

In the CMP polishing process, the utilization of additives to modify the direct mechanical action of abrasive particles on the workpiece or adjust the pH value of the polishing liquid contributes to enhancing the surface quality, alongside optimizing the shape and size of abrasive particles. Two-dimensional nanomaterials with super lubricity, such as graphene, MXene, and hexagonal boron nitride (h-BN), are employed to penetrate the contact surface, smooth sharp edges, and mitigate abrasive wear due to interlayer sliding and low shear stress [41]. In an effort to reduce friction in the contact area, alter the motion of the abrasive from sliding to rolling, achieve super-lubrication in the CMP process, and prevent excessive surface damage caused by an irregular CeO_2_ abrasive, J. Liu et al. incorporated h-BN as a lubricant along with a CeO_2_ abrasive. This approach resulted in the green and effective CMP polishing of fused quartz, achieving surface roughness of 0.124 nm, a maximum MRR of 31.92 μm/h, and a damage thickness of only 2.7 nm [42]. Additionally, the material removal technique is influenced by the polishing fluid environment where abrasive particles are situated. The pH regulator, as an additional component of the CMP polishing fluid, significantly impacts the efficiency of material removal and processing quality. The traditional alkaline polishing fluid, owing to its poor removal efficacy, cannot be employed in industrial production [32]. Researchers have addressed this challenge by developing a novel acidic SiO_2_ slurry, which not only increased the removal rate by 900% compared to the conventional alkaline slurry but also achieved ultra-low surface roughness of Ra 0.193 nm and a high MRR of 10.9 μm/h on a fused silica substrate [27]. Y. Zhou et al. conducted CMP cycle polishing on molten quartz using asphalt plates and a cerium oxide (CeO_2_) acid polishing liquid with HNO_3_ added. The result was an extremely smooth surface with roughness of 0.165 nm. They also observed that the mechanical collisions and friction of abrasive particles, along with the chemical reaction of the slurry composition, contributed to the degradation of the asphalt’s characteristics. As a consequence, the rate of material removal declined, and the roughness of the polished surface increased [43]. This underscores the impact of changes in various parameters of the polishing pad during the CMP processing process on the outcomes. Therefore, finding a polishing pad with stable parameters or developing a practical method to adjust for changing polishing parameters is crucial in achieving ultra-smooth surfaces in CMP processing.

Various composite processing techniques have been developed to address the need for improvements in the material removal rate of the atomic-level removal method in chemical–mechanical polishing. These techniques include electrochemical–mechanical polishing, photocatalytic-assisted chemical–mechanical polishing, and ultrasonic-assisted chemical–mechanical polishing. In electrochemical–mechanical polishing, both the anodic oxide layer and the oxidized surface of the workpiece are concurrently removed by the paste. B. Gao et al. achieved an MRR of approximately 2.3 μm/h and surface roughness (Ra) of 0.449 nm without scratches when applying electrochemical–mechanical polishing to SiC under equilibrium conditions [44]. However, challenges arise in regulating elements like the slurry’s pH and concentration during the polishing reaction process, leading to the inadequate stability of material removal. To enhance the efficiency of achieving a smooth surface, researchers have developed a pulp-less electrochemical polishing method that combines a fixed soft abrasive and anodic oxidation. This approach increased the MRR to 13.6 μm/h, with the surface roughness dropping to 0.37 nm, suggesting that slurry-free electrochemical mechanical polishing holds broad application prospects, particularly in SiC wafer manufacture [45].

**Figure 4:**
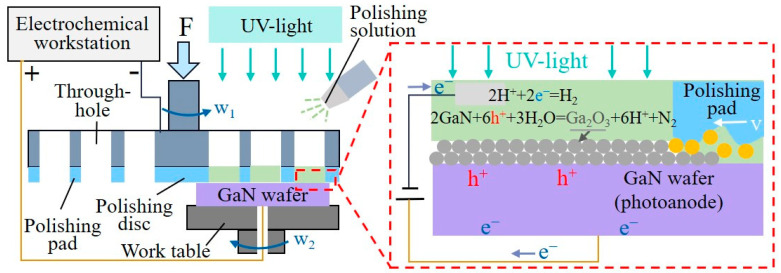
Illustration of the device and principle in photoelectric-assisted chemical–mechanical polishing [62].

Similarly, photocatalytic-assisted chemical–mechanical polishing is an innovative technique combining photocatalytic oxidation and abrasive polishing, utilizing external energy augmentation for surface modification to improve the polishing efficiency and quality [46]. Z. Ye et al. investigated the effects of the UV power and polishing liquid temperature in photocatalytic-assisted chemical–mechanical polishing. They found that increasing the polishing liquid’s temperature and UV LED power significantly increased the MRR and raised the amount of ·OH in the liquid. Under ideal process conditions, a 4H-SiC ultra-smooth surface with surface roughness of Ra 0.0586 nm was achieved [47], although the MRR was 352.8 nm/h. Additional research by W. Wei et al. examined the impact of the catalyst, pH value of the polishing solution, and mass fraction of oxidizer on GaN polishing. The surface roughness dropped to 0.11 nm RMS, and the removal rate of GaN increased to 502.4 nm/h with the addition of UV light and a photocatalyst (SnO_2_) [48]. Starting with the most significant abrasive particles in the polishing slurry, W. Wang et al. combined photocatalysis with a mixed abrasive paste composed of Al_2_O_3_ and ZrO_2_. The oxidation rate was accelerated under ultraviolet irradiation because the ZrO_2_ particles’ electronic transition facilitated the breakdown of H_2_O_2_ into ·OH. Following processing, the surface roughness of SiC was Ra 0.489 nm, and the MRR was further improved to 694 nm/h. With the aim of further enhancing the rate of surface modification, photoelectric-assisted chemical–mechanical polishing was developed as a response to the limited gains in polishing efficiency achieved by merely adding ultraviolet energy [49]. Figure 4 displays the polishing apparatus employed in this technique. Utilizing two types of external energy, the method achieved prepared GaN with internal surface roughness of Ra 0.067 nm and an MRR of up to 1.2 mm/h on the GaN surface—an order of magnitude higher than in the traditional technology. Building upon this, Y. He et al. extensively investigated the impact of pressure and various slurries on photochemical–mechanical polishing [50]. The findings revealed that solely increasing the mechanical action was insufficient to expedite material removal, emphasizing the interplay of both mechanical and chemical factors in the MRR of the SiC wafer. With the application of a slurry and improved process settings, SiC ultra-smooth surfaces with surface roughness of Ra 0.247 nm were produced, and the MRR reached 1.18 µm/h.

The ultrasonic-assisted processing of hard and brittle materials, in addition to the aforementioned auxiliary methods, holds considerable promise [51]. W. Xu et al. presented ultrasonic-aided chemical–mechanical polishing, capable of removing material at a rate of 22.8 mg/h—twice as fast as regular CMP [52,53]. Experiments demonstrated that the addition of ultrasonic vibration increased the workpiece surface’s chemical reaction rate, enhanced the number of effective abrasive removal processes [54], lengthened the effective polishing trajectory of a single abrasive, and successfully reduced sapphire’s surface roughness to 0.083 nm. Moreover, the wise selection of abrasives and dispersants further increased the processing quality and efficiency [55,56]. While the model proposed by J. Luo et al. considered the plastic microcontact between abrasives, silicon wafers, and pads, it overlooked the impact of ultrasonic vibration on the abrasive’s kinetic energy [57]. H. Zarepour et al. created an ultrasonic machining model based on indentation fracture theory because they believed that abrasive particles might remove material by striking the workpiece surface through ultrasonic vibration [58]. This foundation allowed for the clarification of the mechanism of the ultrasound-assisted chemical–mechanical polishing of sapphire using a material removal rate model based on two-body wear and abrasive impact [59]. The two-body wear mode was primarily used to control the polishing pace in order to produce a higher MRR, and high-frequency ultrasonic vibration was used to drive the abrasive to strike the sapphire surface. The combined action of the two removal mechanisms produced an ultra-smooth sapphire surface with surface roughness of Ra 0.07 nm. The advancement of chemical–mechanical polishing technology with ultrasonic assistance has been facilitated by a comprehensive understanding of the material removal mechanism. It also comprises friction chemical mechanical polishing [60], atomization chemical mechanical polishing [61], and other techniques in addition to the ones mentioned above. Since this study’s primary focus is on the ultra-smooth processing effect of atomic-level material elimination, they are not be covered in detail.

CMP stands out as a pivotal technique in achieving ultra-smooth surfaces, capable of removing material at the atomic level through a combination of chemical reactions with surface atoms and the mechanical action of micro-abrasive particles. While abrasive particles with larger sizes and sharp edges can yield a higher MRR, the resultant surface tends to be rough due to the inherent conflict between maximizing material removal and ensuring surface quality. Conversely, abrasive particles characterized by smaller sizes and high roundness can trade a lower MRR for a smoother surface. Ongoing research is delving into the intricacies of the particle size and shape. By adjusting parameters such as pH regulators, chemical additives, and other process variables, regular polyhedral particles, striking a balance between spherical and sharp surfaces, can optimize the interplay between the MRR and surface roughness. This nuanced approach contributes to the continual advancement of the CMP ultra-smooth surface processing capacity.

### 3.2. Elastic Emission Machining

**Figure 5:**
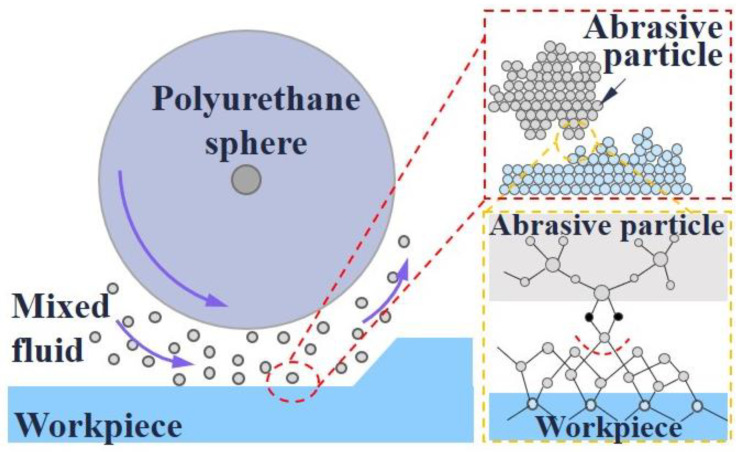
Schematic diagram of the working principle of elastic emission machining technology [64].

The inception of elastic emission machining (EEM) technology is credited to Mori of Osaka University in Japan. EEM employs a rotating polyurethane ball to generate hydrodynamic pressure, causing abrasive particles to impinge on the workpiece surface at an approximately horizontal angle, leveraging weak atomic bonding forces present on surface irregularities. Furthermore, the chemical activity of the abrasive particles interacts with the substance’s atoms, facilitating atom-level removal. The polishing principle is depicted in Figure 5 [63,64].

More than a decade elapsed after Mori’s 1976 proposal of the EEM process, and few investigations in this area were published. It was not until 1988 that Mori and colleagues, through testing, discovered the superior surface quality produced by the EEM approach compared to the chemical etching method [65,66]. Y. Su et al.’s investigation into the material removal mechanism during EEM processing revealed an inverse relationship between the MRR and the liquid film thickness distribution between the tools and workpieces. Abrasive particles remove materials by breaking the chemical bonds between surface atoms and sublayer atoms [67]. The material removal method of abrasive particles in jet fluid was explored based on the angle of abrasive deformation and cutting wear [68]. L. Zhang et al. utilized hydrodynamic lubrication theory to further examine changes in shear stress and pressure gradient in liquid films. Through experimentation, they qualitatively confirmed the impact of the process parameters on the polishing surface quality [69]. Shear, rolling and fatigue are the three main impacts resulting from abrasive particles colliding with the workpiece surface at varied angles and rotation rates due to the different energy imparted [70]. The particle impact wear theory posits that the overall effect of the nanoparticle on the surface roughness peak satisfies the removal quantity expression of the Gaussian distribution. However, during the actual processing phase, the final material removal will also be impacted by the collisions of the nanoparticle with the workpiece and the polishing wheel, so these factors must be considered when constructing the material removal model. D. Wen et al. employed computational fluid dynamics (CFD) to investigate the impact of the polishing disc’s rotation on the abrasive particle velocity and collision angle [71]. Based on this, the researchers integrated particle contact mechanics, rough surface fractal theory, computational fluid mechanics, and an erosion process to create a material removal model of fused silica glass deformation and cutting wear [72]. After processing, the PV value was 9.48 nm, and the surface roughness was 0.642 nm. High prediction accuracy was achieved, with the greatest error of the anticipated PV and Ra values being 3.7% and 5.6%, respectively.

**Figure 6:**
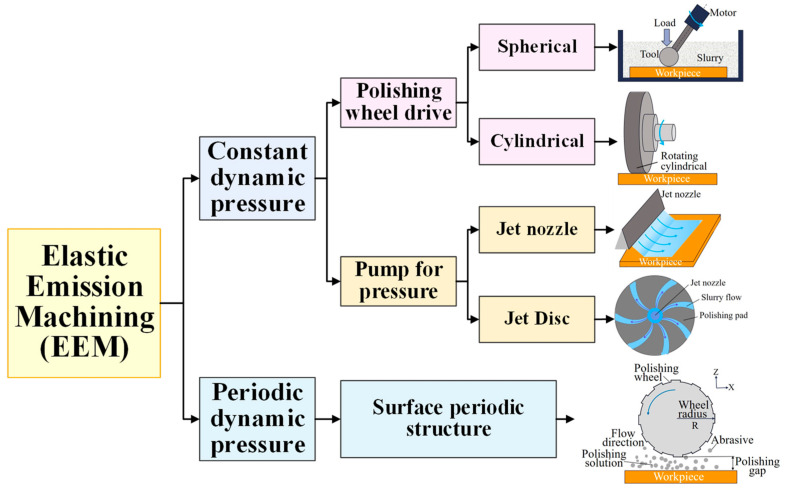
Classification of elastic emission machining technologies.

Process parameters and processing devices undergo continuous updates and development based on an in-depth analysis of the EEM material removal mechanism. The suggested hydrodynamic pressure ultra-smooth machining method is also included in the category of EEM, based on the EEM principle. These methods are categorized based on the dynamic pressure characteristics of the polishing fluid generated during the machining process, as illustrated in Figure 6. In traditional EEM processing equipment, the steady hydrodynamic pressure produced by the polishing wheel is typically used to process the surface. Initially, Y. Mori et al. employed a cross spring to regulate the machining clearance of the polishing ball. However, the characteristics of the spring could be changed, and the device control’s accuracy and response time were insufficient [73]. The researchers further investigated the mechanism by which the machining clearance influences the roughness. The findings demonstrated that the clearance affected the stability and dependability of the removal function, which, in turn, affected the surface roughness. A machining gap of 25 μm allowed for the creation of an extremely smooth surface with Ra 0.116 nm [74]. While controlling the clearance can yield an ultra-smooth surface, the unstable radial runout during the polishing wheel’s rotation can replicate equipment errors on the surface, resulting in ripples or fine lines. This can exacerbate the surface roughness, making it more challenging to meet application requirements [75]. Using an elastic emission processing device with dual rotation of polishing wheels and tools, Q. Li et al. reduced the surface roughness of quartz glass from 0.969 nm RMS to 0.0801 nm RMS. They also processed monocrystalline silicon to a surface roughness value of 0.151 RMS, achieving atomic-level removal [76].

To achieve an ultra-smooth surface, the active ingredients in the polishing liquid undergo a chemical reaction with the workpiece surface. The reaction products are then removed from the surface using hydrodynamic pressure. O. Reynolds laid the theoretical groundwork for hydrodynamic pressure acting on the material removal process by proposing a differential equation related to hydrodynamic lubrication theory [77], revealing the mechanism of the hydrodynamic pressure generation of fluid and proposing a three-dimensional fluid dynamics model based on tribology and fluid corrosion theory [78,79]. Figure 6 illustrates how hydrodynamic pressure is produced. There are two primary forms of polishing wheels: spherical and cylindrical. The cylindrical polishing wheel falls under the line contact category and is suitable for processing flat surfaces because its lowest position is the same distance from the workpiece surface. In contrast, the point-contact spherical polishing wheel, with its different shape, is more versatile and can be used to process the surface of monocrystalline silicon. The surface roughness of silicon was reduced from 0.737 nm RMS to 0.175 nm RMS, and an ultra-smooth surface of 0.155 nm RMS was achieved for the handling of glass cylindrical surfaces [80]. Additionally, a nozzle-type machining device for pressurized pump pressure has been developed rapidly based on the idea of hydrodynamic pressure. Using a focused flow nozzle, T. Yoshinori et al. controlled the material removal rate and removal range, significantly enhancing the capacity of nozzle dynamic pressure processing to modify the form while preserving the quartz surface roughness at 0.518 nm RMS [81]. Researchers have also developed rectangular nozzles and treated monocrystalline silicon with agglomerated and spherical silica particles, resulting in surface roughness values of 0.141 nm RMS and 0.098 nm RMS, respectively [82,83]. Further improvements were made by Z. Ma et al., who processed fused silica using a porous micro-jet device, reducing the surface roughness from 1.02 nm to 0.56 nm [84]. J. Wang et al. achieved surface roughness of 0.0726 nm by using an ion beam iterative polishing technique and a porous micro-jet slotting polishing tool, although the iterative process required more time and resources [85]. Traditional hydrodynamic polishing (DHDP) of polishing discs, converting straight grooves into curved grooves, has also contributed to the exploration of elastic emission polishing technology [86]. X. Jiang et al. optimized the process parameters to process fused silica glass in an ultra-smooth manner, reducing the surface roughness Ra to 0.642 nm [72].

**Figure 7:**
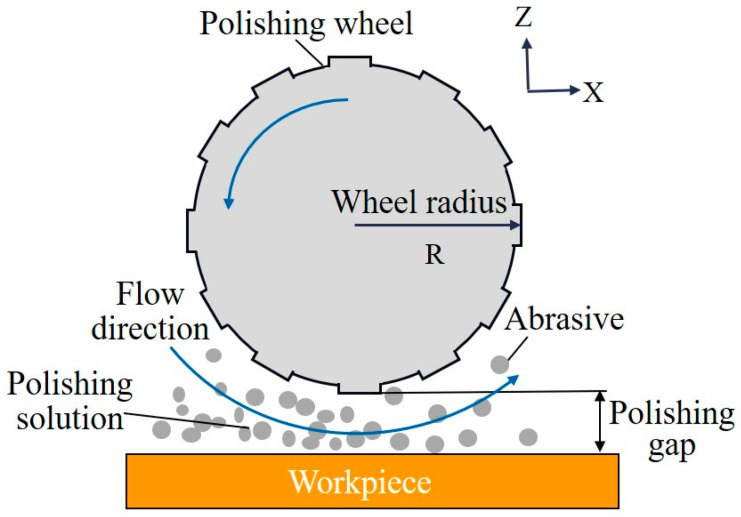
Principle of linear hydraulic polishing [89].

In contrast to the constant dynamic pressure method discussed above, a regularly changing fluid dynamic pressure can be achieved by incorporating microstructures onto the typical polishing wheel’s surface. S. Ji et al. introduced groove structures to conventional cylindrical polishing wheels and, through comparative research, found that the groove polishing tools outperformed their conventional cylinder counterparts in terms of removal rates and roughness rates under identical processing conditions [87]. Although achieving ultra-smooth surface processing remains elusive, the microstructure of polishing wheels proves beneficial in reducing surface roughness. Building upon this, Z. Zheng et al. conducted further comparisons involving rolls featuring wedge, parabolic, and rectangular microstructures [88]. They observed that rolls with rectangular microstructures were capable of generating more uniform hydrodynamic pressure. Figure 7 illustrates that when the rectangular microstructure was identified, 12 rectangular rolls generated pronounced pressure, surpassing other roll types [89]. Treating the K9 glass workpiece with the optimal process parameters resulted in a noteworthy reduction in surface roughness (Ra) from 45.41 nm to 0.91 nm, showcasing the effectiveness of the linear hydrodynamic pressure ultra-smooth machining technique. W. Fu et al. delved into the effects of the roll size, rotational speed, and polishing gap on hydrodynamic pressure and viscous shear stress, based on earlier research. Subsequently, they optimized the process parameters, achieving precise control over the surface roughness (Ra) of K9 glass at 0.21 nm and realizing ultra-smooth surface machining [90].

While EEM exhibits versatility in various processing techniques, its primary role lies in stabilizing the generation of fluid dynamic pressure and directing its flow. The hydrodynamic pressure in EEM is significantly influenced by the surface quality of the polishing wheel and radial runout, given the stringent requirements that EEM imposes on the machining gap. Consequently, deviations from the design goal may occur in the machining results. Thus, minimizing the adverse effects of these factors becomes imperative to enhance the quality of EEM. The spherical polishing wheel’s point contact mode, suitable for processing intricate surface shapes, contrasts with the cylindrical and rectangular nozzle’s line contact mode, which excels in flat polishing, leveraging guide fluid dynamic pressure and workpiece surface contact. While EEM can attain ultra-smooth surface processing with a high level of surface roughness, its processing efficiency remains constrained, and challenges persist in addressing the demanding motion accuracy requirements of the equipment.

## 4. Atomic-Level Removal of Materials under Energy Beam Effect

### 4.1. Ion Beam Finishing

**Figure 8:**
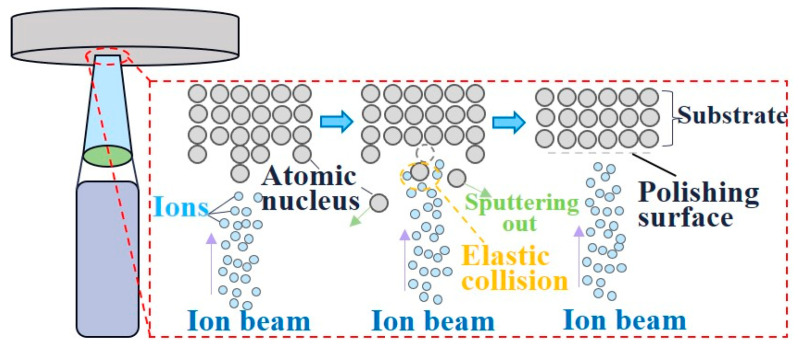
Principle of ion beam finishing [92].

Ion beam finishing (IBF) involves the shaping and acceleration of ions generated through ionization, subsequently directing them to bombard the workpiece’s surface. This process induces intricate collisions between the ions and the atoms of the workpiece [91]. The kinetic energy possessed by the ions is transferred to the surface atoms of the material. Figure 8 illustrates the occurrence of sputtering, where the energy acquired by the surface atoms surpasses their binding energy, achieving the precise removal of material at the atomic level.

The inaugural application of ion beam technology in optical processing took place in 1965 when American researchers utilized ion beams for the polishing of optical glass [93]. The Kaufman-type ion source, capable of providing ion beams suitable for the required ion energy range essential for processing optical components, was not available until the 1970s [94]. A study examining the factors influencing ion beam machining revealed that the optimization or degradation of machining outcomes significantly varies with the initial surface roughness [95]. These trends eventually converge to a specific roughness level. Fused silica with low initial surface roughness experiences gradual deterioration from 0.40 nm to 0.64 nm throughout processing, while there is a decrease in surface roughness from 1.54 nm to 0.49 nm [96]. Analyzing the microscopic morphological evolution induced by ion beam finishing (IBF), W. Liao et al. highlighted the substantial impact of the surface/subsurface damage created during preprocessing on the ultra-smooth manufacturing process [97]. Since IBF is highly sensitive to variations in the microscopic properties of materials, ion beam processing is conducted on high-quality fused silica surfaces following chemical mechanical polishing as a pretreatment step. The result is an ultra-smooth surface with roughness of 0.06 nm RMS, a significant benchmark for the choice of the preceding ion beam ultra-smooth machining procedure. To fully comprehend the influence of initial surface imperfections on the final polishing outcome, it is imperative to examine the evolution of the original surface flaws under ion beam irradiation. Surface and subsurface flaws can be eliminated or passivated when the ion incidence angle is between 0° and 30°; however, as the angle increases, the change in nanostructure leads to a worsening trend in surface roughness. Thus, by polishing fused silica with an incidence angle of 0°–30°, the surface roughness can be reduced to 0.416 nm [98], eliminating surface imperfections and impurities while enhancing the damage threshold of optical elements. Further investigation led to the efficient surface smoothing of machined fused silica by optimizing the incidence angle to a range of 15°–30° [96]. Considering the possibility of secondary flaws persisting after the process, the combination of dynamic chemical etching and ion beam polishing becomes relevant [99]. After processing, fused silica’s surface roughness approaches 0.3 nm RMS, significantly raising the damage threshold.

### 4.2. Plasma Polishing

Plasma, acknowledged as the fourth state of matter alongside solids, liquids, and gases, is a quasi-neutral gas composed of charged and neutral particles exhibiting collective behavior. To facilitate material removal and processing, inert gases such as argon or helium ionize when subjected to an external high-frequency electromagnetic field, forming a macroscopic electroneutral and highly active plasma. This activated state transforms the reaction gas into chemically active particles, subsequently sprayed onto the surface of the workpiece. On contact, these particles undergo chemical reactions with the surface materials, achieving surface modification or resulting in the formation of volatile products [100].

**Figure 9:**
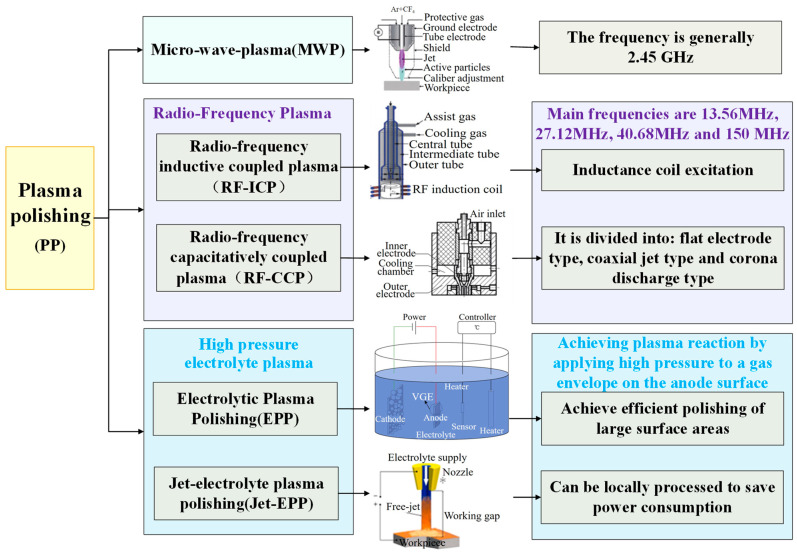
Classification of plasma polishing.

Plasma polishing (PP) is categorized under micro-wave plasma (MWP) based on the mode of plasma excitation, alongside radio-frequency plasma [101,102] and high-voltage electrolyte plasma polishing (EPP) [103]. Recent advancements in plasma processing have seen researchers delve deeper into the polishing principles and apparatus. Notably, the achievement of ultra-smooth material surface processing has been realized through the implementation of innovative polishing tools and techniques. Figure 9 illustrates the classification of plasma processing techniques.

Amidst the ongoing comprehensive exploration of plasma removal mechanization, R. Yi et al. introduced a comprehensive metal finishing technique centered on isotropic etching polishing (IEP) [104]. This concept laid the groundwork for the combination of plasma polishing and a plasma-based isotropic etching approach (Plasma-IEP) by R. Li et al., employed in polishing synthetic quartz with inner surface roughness of only 0.145 nm [105]. However, a limitation in surface quality improvement was encountered due to the overlap of isotropic etching pits during processing. To address this challenge, H. Deng et al. achieved a further reduction in surface roughness by processing isotropically polished CeO_2_ stone with a surface mixture of PAP and resin without harm. The surface roughness of CVD-SiC rapidly decreased to 0.69 nm RMS after a 10-min processing period [106]. Soft grinding heads and plasma-assisted polishing have been demonstrated to be crucial in reducing the CVD-SiC surface roughness. Plasma-based atom selective etching (PASE), targeting microscopic surface atoms by selectively eliminating Si atoms with more suspended surface bonds at high temperatures, contrasts with macroscopic isotropic etching and accomplishes polishing [107]. This method provides a robust theoretical foundation for the achievement of ultra-smooth surfaces at the microscopic level. Processing gallium nitride (GaN) using PASE for two minutes resulted in a high MRR of 93.01 µm/min, rapidly reducing the surface roughness from Sa 135.8 nm to 0.527 nm [108] when compared to conventional chemical mechanical polishing procedures.

**Figure 10:**
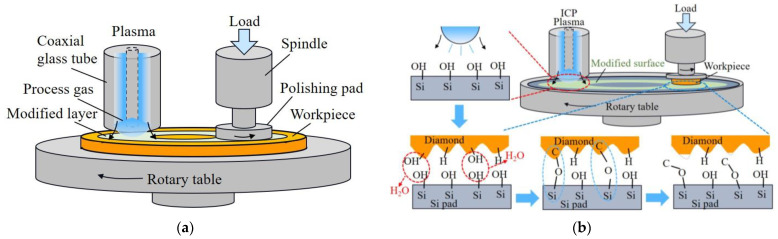
Illustration of plasma-assisted polishing device: (**a**) direct surface modification [109], (**b**) indirect surface modification [114].

Plasma-assisted polishing (PAP) stands out as an efficient method for surface modification, particularly in enhancing the polishing efficiency of challenging materials such as silicon carbide and diamond. Two distinct approaches to material removal are employed in this process. The first method involves direct surface modification, wherein the workpiece’s surface is promptly coated with plasma, resulting in the formation of a modified layer. Figure 10 illustrates the modification of the SiC workpiece’s surface using plasma, followed by the achievement of an ultra-smooth surface with roughness of 0.28 nm RMS through subsequent soft abrasive polishing [109,110]. While PAP exhibits potential as an effective and safe technique for the machining of SCD substrates, the significance of the sliding speed and polishing pressure in the material removal process remains uncertain. Addressing this, N. Liu et al. investigated the impact of the pressure and sliding speed on diamond material removal during PAP polishing [111]. Their findings revealed that higher polishing pressure or sliding speeds between the polished plate and substrate could enhance the MRR, and controlled pressure classification could yield an ultra-smooth surface devoid of a damaged layer, achieving final surface roughness of 0.3 nm, with an MRR reaching as high as 5.3 µm/h. However, the use of sophisticated processing equipment and vacuum treatment conditions poses challenges for industrial applications. Another technique involves indirect surface modification removal, where the ·OH radical-assisted diamond polishing method facilitates abrasion-free single crystal diamond (SCD) polishing [112,113]. By polishing SCD using a revolving iron plate submerged in hydrogen peroxide, the surface roughness can be reduced from Ra 1.87 nm to 0.13 nm. H. Luo et al. proposed an inductively coupled plasma (ICP)-enhanced SCD polishing process based on the principle of using a (·OH) modified monocrystalline silicon polishing plate generated by atmospheric pressure plasma. In this process, material is efficiently removed from the workpiece surface via a chemical reaction between the polishing pad and the surface, achieving a remarkably smooth surface with roughness of Ra 0.26 nm [114].

## 5. Application of Flexible Contact Wear Processing Technology

**Figure 11:**
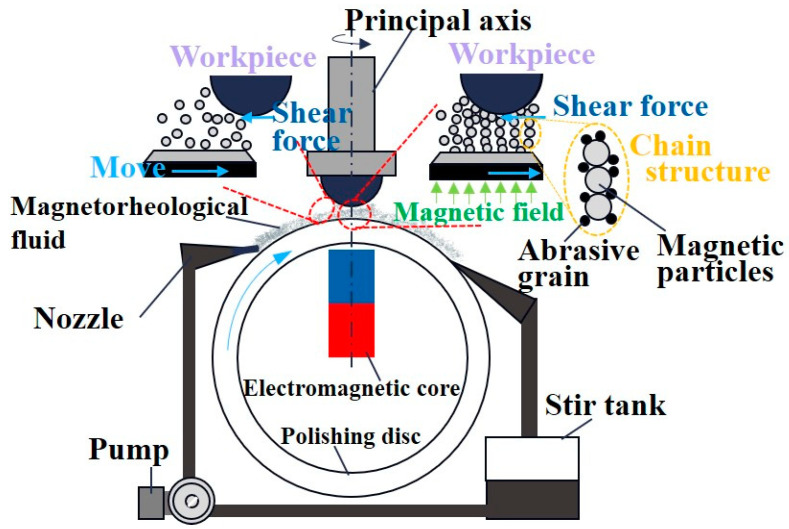
Schematic diagram of the working principle of magnetorheological finishing [117].

At a microscopic level, the fundamental process underlying flexible contact wear and polishing removal involves the removal of atoms or atomic clusters from the workpiece’s surface material through the interaction with numerous tiny particles. A prominent technique in this realm is magnetorheological finishing technology (MRF), which represents a comprehensive approach to achieving ultra-smooth surface processing, integrating analytical chemistry, fluid dynamics, and electromagnetism. Initially proposed by the Belarusian scholar Kordonski in 1988, MRF was further developed and refined by academics at the University of Rochester in the United States [115]. MRF utilizes the properties of magnetically sensitive particles in a magnetorheological fluid, which, under the influence of a magnetic field, aggregate into strings. These strings, in turn, entrain abrasive particles, facilitating their action on the workpiece surface through a combination of shear stress and normal stress. This process, driven by the combined action of water molecules and surface hydrolysis, achieves the removal of single atoms or atomic groups [116]. Under specific process conditions, MRF has the capability to reduce the surface roughness to sub-nanometer levels. Figure 11 provides an illustration of its operational principles.

**Figure 12:**
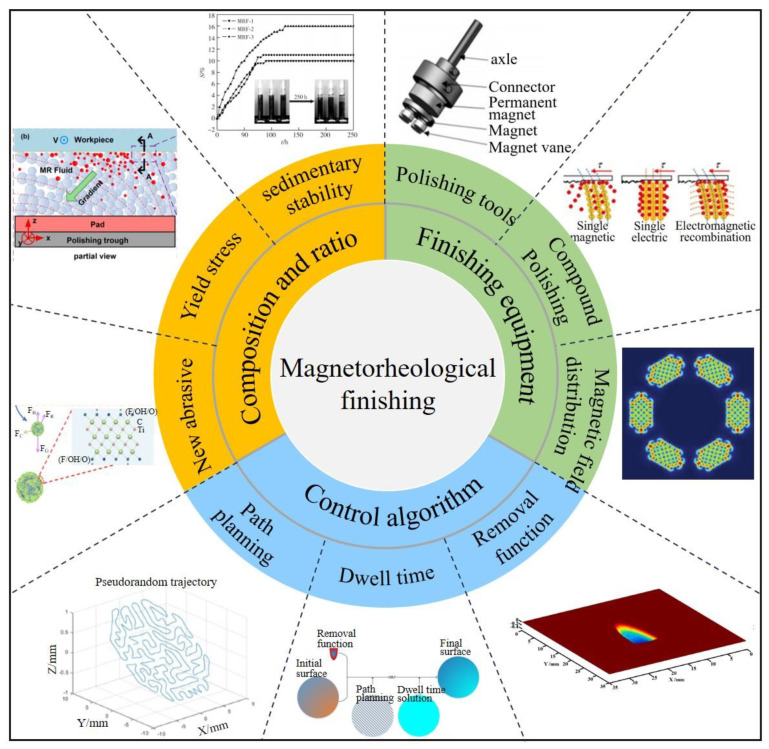
Main research content of magnetorheological finishing.

To enhance the precision of magnetorheological deterministic machining, recent research has predominantly concentrated on refining magnetorheological polishing technology, specifically addressing the composition and proportion of the magnetorheological fluid, advancements in processing equipment and techniques, and the development of control algorithms for polishing heads. The complexity of influencing factors in the magnetorheological polishing process necessitates substantial and stable control. Figure 12 illustrates that the attainment of ultra-smooth material surface processing is feasible through the meticulous optimization of crucial elements within the polishing process.

A magnetorheological fluid, a critical component in the magnetorheological finishing (MRF) process, typically constitutes a non-colloidal suspension comprising four key elements: additives, a base loading fluid, abrasive particles, and magnetically sensitive particles. The primary characteristics of magnetorheological fluids include the shear yield stress, magnetic properties, and settlement stability. Researchers, both domestically and internationally, have endeavored to enhance the removal efficiency and settlement stability of magnetorheological fluids by optimizing these four components. However, a common challenge arises as magnetically sensitive particles in standard magnetorheological fluids hinder abrasive particles from making contact with the workpiece surface. To address this, M. Amir et al. developed magnetic nanoparticles with dual characteristics, which were employed in the magnetorheological polishing of the BK-7 substrate surface [118]. This innovation resulted in an 88.14% increase in Ra on the surface, while the use of recyclable magnetic nanoparticles contributed to cost reduction by resolving issues related to magnetorheological fluid pollution. Nevertheless, limitations emerged due to the small contact area between the magnetic flux variant and the workpiece surface, attributed to the grinding head’s size constraints and the magnetic field’s intensity. Subsequently, the development of cluster magnetorheological polishing has rapidly evolved [119,120]. This method significantly augments the polishing efficiency by transforming single-point polishing into multi-point polishing through the strategic insertion of permanent magnets into the polishing disc in a predetermined pattern. By enhancing the contact area between the magnetic fluid version and the workpiece, this technique leverages a single-sided magnetic field that is stronger and features fewer magnetic poles [121]. In a mere 60 min, the surface roughness (Ra) dramatically decreased from 1979.154 nm to 0.544 nm during the molten silica polishing procedure. Achieving an MRR of 1.38 mm^3^/min, this represents a 3.8-fold improvement over the current procedure.

**Figure 13:**
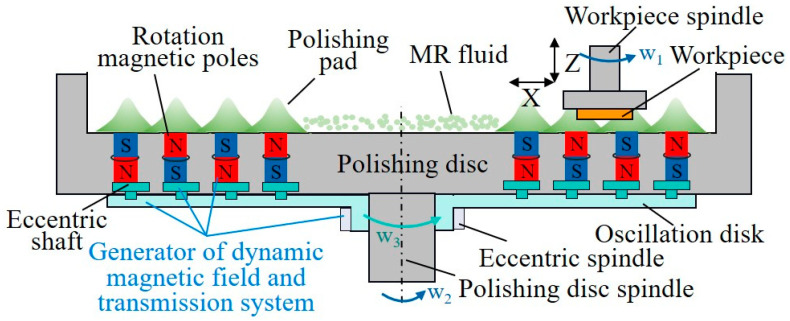
Schematic diagram of dynamic magnetic field cluster magnetorheological finishing device [122].

The insufficient uniformity of the magnetic field in the machining region during static cluster magnetorheological polishing results from the varying length of the machining track at different workpiece positions and the uneven distribution of the magnetic field along the track. S. Sun et al. employed dynamic magnetic field cluster magnetorheological polishing, as illustrated in Figure 13. Processing a 6-inch silicon wafer for 120 min, the device achieved a final surface roughness value of Sa 0.44 nm on average [122]. However, the wheeled magnetorheological machining device offers superior advantages in terms of the workpiece’s shape flexibility compared to the unsuitability of the magnetic field cluster on the plane for surface cutting. To overcome this limitation, W. Zhang et al. enhanced the tool, creating two polishing belts based on the conventional single-ribbon magnetorheological polishing wheel. This modification allowed the polishing of large, curved workpieces. The surface roughness of the fused silica optical device measured 0.616 nm RMS after processing, achieving a volume removal rate of 2.03 μm^3^/min [123]. Beyond intensifying the magnetic fields, the control algorithm of polishing tools is pivotal for efficient and deterministic magnetorheological machining. An improved two-level Iterative Closest Point (ICP) matching approach proposed by T. Zhou et al. [124] addresses the challenge of the high efficiency and precise self-positioning of symmetric aspherical workpieces. This method achieves positioning accuracy greater than 9 μm, with an average positioning time of 7.3 min. Concerning magnetorheological polishing path planning, an appropriate algorithm can enhance the uniformity of the Gaussian distribution removal function on the workpiece surface and mitigate the influence of the edge effect by extending the edge region [125,126].

A valuable strategy to enhance the quality and efficiency of magnetorheological polishing involves magnetic flow variable pressure composite polishing. The surface structure or vibration of the polishing disc can generate fluid dynamic pressure, and the outcomes of various dynamic pressure sources also vary. In pursuit of ultra-smooth workpiece polishing, W. Peng et al. employed a sequential combination of magnetorheological finishing (MRF) and hydrodynamic pressure polishing, resulting in surface roughness values of 0.268 nm RMS for middle frequencies and 0.163 nm RMS for high frequencies [127]. However, the efficiency of this sequential combination during device conversion is comparatively lower. The integration of fluid dynamic pressure polishing and magnetorheological polishing is achieved through a variable gap dynamic pressure magnetorheological polishing method combined with *Z*-axis squeezing vibration. This approach enhances the stability and efficiency of polishing, ultimately reducing the surface roughness Ra of the sapphire workpiece from 6.22 nm to 0.31 nm, with an MRR of 5.52 nm/min [128]. A similar principle is employed in ultrasound-assisted magnetorheological polishing, where axial ultrasonic vibration enhances the polishing effectiveness by promoting the renewal of abrasive particles in the gap. The minimal surface roughness achieved after processing sapphire using this method is as low as 0.276 nm, with a maximum relative growth rate of up to 2.068 μm/h [129]. Additionally, introducing periodic wedge-shaped grooves on the rotating polishing disc, coupled with increased *Z*-axis vibration, contributes to fluid dynamic pressure generation [130]. The primary purpose of these grooves is to vary the distance between the workpiece and the polishing pad regularly, enhancing the effectiveness of magnetorheological polishing. In a comparative study of grooves, wedges, circular microporous arrays, and smooth surface polishing discs, B. Luo et al. found that the microporous array polishing disc, with a systematically designed structure, achieved a rough surface of 0.104 nm for sapphire ultra-smooth machining under optimal process conditions [131,132,133].

**Figure 14:**
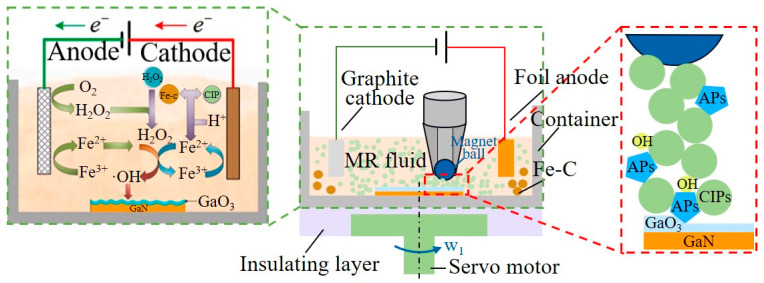
Illustration of electro-Fenton reaction-assisted magnetorheological finishing device [135].

While chemical mechanical polishing can yield fine surface quality, its drawbacks include low processing efficiency and an unpredictable chemical corrosion rate and depth. Conversely, magnetorheological polishing, when effectively assisted by the Fenton reaction induced by Fe^2+^ and H_2_O_2_, can produce an ultra-smooth surface. Following the Fenton reaction on the oxidized SiC surface, H. Liang et al. utilized magnetorheological polishing to eliminate the surface oxide layer, resulting in an ultra-smooth surface with roughness of Ra less than 0.10 nm and an MRR of up to 2.4 mg/h [134]. J. Pan et al. tackled the challenges associated with the homogeneous Fenton reaction, such as harsh conditions, rapid oxidant consumption leading to reduced reaction activity, and the difficult separation and recovery of the resulting iron sludge [135]. They initiated the Fe^2+^ and H_2_O_2_ process through electrolytic oxidation–reduction, leveraging the electro-Fenton reaction to assist in magnetorheological polishing. Figure 14 illustrates the experimental setup, marking a significant advancement in the Fenton reaction-assisted magnetorheological polishing technique by addressing the issue of uncontrollably high reaction rates and maintaining the reaction stability throughout the machining process.

Magnetorheological polishing primarily relies on the cutting action of flexible contact to eliminate surface atoms or atomic clusters, differing from other techniques for ultra-smooth surface processing that directly engage with surface atoms. Consequently, the processed surface may exhibit micro scratches. Despite this, magnetorheological polishing presents notable advantages in terms of processing efficiency and adaptability to the shape and size of the workpiece. Furthermore, ongoing innovation and refinements in process methods have successfully achieved surfaces with sub-nanometer-level roughness, even with the relatively substantial material removal.

## 6. Comparison of Technical Properties of Ultra-Smooth Surface Processing

The demands for precision and quality in optical systems are approaching the physical limit, driven by the rapid development of large-scale, precise scientific instruments. Simultaneously, the escalating requirements for workpiece surface quality accompany this progression. High-quality wafer surfaces contribute to the stability of integrated circuits in chip manufacturing, and ultra-smooth surfaces, including fused silica, sapphire, monocrystalline silicon, and silicon carbide, find extensive application in optical, electronic, and various other fields. These ultra-smooth surfaces significantly enhance the reflectivity and damage thresholds of optical components.

A comparative analysis of several ultra-smooth surface processing techniques reveals that, although the techniques differ, the key avenues to enhance their performance include (1) investigating the optimal parameters for the processing of specific materials using the technology, encompassing the processing path, speed, abrasive particle size, and environmental conditions; (2) improving the accuracy of the model algorithm’s predictions by optimizing and validating the material removal model and control algorithm for the given processing technique; (3) elevating existing processing techniques, such as optimizing local polishing devices and implementing the composite processing of various techniques, to enhance the surface quality by leveraging each of their advantages; (4) proposing specific solutions to address potential challenges in the machining process, including edge effects, thermal accumulation, surface oxidation, and workpiece contamination.

**Figure 15:**
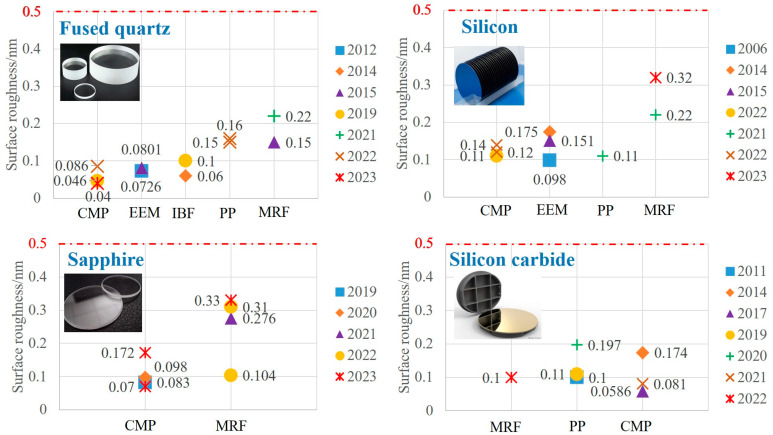
Comparison of machining properties of four types of materials by different ultra-smooth surface machining techniques.

This section provides a summary of the surface roughness outcomes for fused silica, sapphire, monocrystalline silicon, and silicon carbide following treatment with diverse processing methods. The surface processing techniques discussed include CMP, EEM, PP, IBF, and MRF, with surface roughness being a crucial parameter in evaluating the performance of these methods. Illustrated in Figure 15 (note: some of these techniques may not have been utilized in material processing, or their surface quality may not significantly differ from the current quality data depicted in the figure; the primary focus of the composite processing approach is the resultant roughness that it generates), a horizontal comparison of the lowest surface roughness for the four materials allows for an assessment of the processing performance of various techniques. This comparative analysis serves as a valuable reference in advancing the development of ultra-smooth surface processing.

Comparing the performance of ultra-smooth surface processing techniques necessitates the use of the same material for evaluation due to the inherent differences in the characteristics of fused silica, monocrystalline silicon, silicon carbide, and sapphire, as shown in the Table 1. The attainment of an ultra-smooth surface is facilitated by a range of processing methods, particularly applicable to materials like fused silica and monocrystalline silicon, characterized by low Mohs hardness and high chemical reactivity. Conversely, materials such as silicon carbide and sapphire, boasting high Mohs hardness and stable chemical properties, pose challenges for effective material removal through chemical reactions and fluid dynamic actions like EEM. Optimal results are achieved when employing specific machining techniques tailored to the material’s properties. Fused quartz glass and monocrystalline silicon can attain surface roughness of less than a nanometer using the techniques outlined in Figure 15. Moreover, certain fused quartz processing methods, including CMP, EEM, and IBF, can even reach sub-nanometer-level roughness. Similarly, CMP processing for silicon carbide and sapphire materials can yield comparable smoothness. Both CMP and EEM demonstrate efficacy in achieving ultra-low surface roughness. Consequently, projects emphasizing ultra-low surface roughness hold significant potential for both chemical–mechanical polishing and elastic emission polishing. Nonetheless, challenges persist, such as maintaining the stability of the polishing fluid during free abrasive CMP processing. The wear of the polishing pad and passivation of abrasive particles in consolidated abrasive CMP also impact the stability of the final material removal. EEM, on the other hand, demands meticulous control over the processing gap, necessitating strict regulation of the polyurethane polishing wheel’s surface roughness and minimizing radial runout.

IBF is commonly employed for precision polishing following pre-polishing stages, leveraging energy transfer to act on surface atoms and achieve lower surface roughness. However, it imposes heightened prerequisites on the initial surface roughness of the workpiece. Plasma polishing, utilized in ultra-smooth surface processing, primarily employs plasma to chemically modify and soften the workpiece surface in a plasma-assisted manner. It combines the flexible contact wear of polishing wheels with abrasive particles for material removal, with plasma directly sprayed onto the workpiece surface, generating volatile products for material removal. Nevertheless, this method may lead to oxidation deposition in the processing area, posing challenges in achieving ultra-smooth processing with extremely low surface roughness. For materials like sapphire, known for its hardness and brittleness, achieving ultra-smooth polishing with minimal surface roughness is best accomplished through CMP and MRF. Both CMP and MRF surpass conventional polishing techniques in achieving superior surface roughness. Upon careful consideration of the processing techniques and quality across different materials, CMP and MRF exhibit notable flexibility in processing materials, contributing positively to the reduction of surface roughness. In comparison, while MRF introduces fine scratches due to flexible contact wear, it demonstrates commendable overall processing efficiency and consistent removal functionality, enabling deterministic processing. The attainment of an ultra-smooth surface in practical processing relies on selecting the appropriate techniques. A comprehensive analysis of various ultra-smooth surface processing techniques allows for a compilation of their distinct advantages and drawbacks, facilitating effective comparisons and serving as a guide for the creative combination of composite processing techniques for ultra-smooth surfaces. Specific details are presented in Table 2.

## 7. Summary and Perspectives

In the rapidly evolving landscape of technology, optical components face increasingly stringent performance demands, particularly in high-precision applications such as lithography machinery and space observation. This trend poses new challenges to optical manufacturing technology, especially in the realm of surface processing techniques. This paper aims to overview and assess the latest advancements in ultra-smooth surface processing technologies, exploring how they meet these elevated standards. We focus on the development of key technologies such as CMP, EEM, IBF, PP, and MRF and analyze their impact on the manufacturing of optical components. The summary and perspectives of this paper are as follows.

(1) The progression of optical components, driven by the escalating performance demands in optical systems such as lithography machines and space observation, poses a formidable challenge to optical manufacturing technology. As these components become integral to systems, notably in the optical–mechanical domain, achieving ultra-low surface roughness, minimizing sub-surface damage, and mitigating residual stress are imperative to align performance with design specifications. Consequently, the field of ultra-smooth surface processing technologies has witnessed significant advancements, continually refining the quality of ultra-smooth surfaces. This paper presents an analysis and synthesis of the principles underpinning CMP, EEM, IBF, PP, and MRF technologies, elucidating their recent progress. The presented comparison discerns the distinctive features and properties inherent in these various processing technologies, offering guidance for the ongoing development of ultra-smooth surface processing technology.

(2) The pursuit of ultra-smooth surfaces has prompted the compositional optimization of numerous polishing methods along with their process parameters. However, techniques focusing on atomic-level material removal have demonstrated advancements in surface quality, albeit constrained by a trade-off between the MRR and surface smoothness. Taking CMP as an illustrative example, the use of larger abrasive particles with sharp edges enhances the MRR but results in rough surfaces. Conversely, opting for smaller, rounder particles yields smoother surfaces at the expense of the MRR. Striking a balance between the MRR and surface roughness necessitates further investigation into the size and shape of wear particles, particularly those transitioning between spherical and sharp forms. Alternatively, the introduction of lubricants can modify the wear particle’s contact state with the surface, thereby augmenting the processing capacity of CMP to achieve ultra-smooth surfaces.

(3) The precision of EEM is susceptible to variations in the hydrodynamic pressure caused by the radial runout and surface quality of the polishing wheel. These deviations can lead to discrepancies between the actual machining results and the intended design. The presence of porosity on the surface of the polyurethane polishing wheel poses challenges in measuring the roughness of the holes, resulting in missing values. Enhancing the accuracy of the polishing wheel’s surface quality evaluation can be achieved by optimizing the process parameters to minimize the number of surface faces or by employing data interpolation for missing spots. Further elevating the rotational speed of the polishing wheel is hindered by its radial runout, impeding the improvement of the EEM processing efficiency. While EEM is capable of achieving an exceptionally smooth surface with a high degree of surface roughness control, its processing efficiency remains limited, and addressing the demanding motion accuracy requirements of the equipment is imperative for future advancements.

(4) Comprehensive research efforts are imperative to gain a thorough understanding of the material removal mechanism in ultra-smooth surface processing. The existing material removal models often make assumptions about the size, shape, and relative velocity of abrasive particles, introducing errors between the model and actual outcomes. To enhance the predictive accuracy of the model, future developments should consider the actual action mechanism of these simplifying elements and guide the optimization of the process parameters. Chemical reactions play a pivotal role in ultra-smooth machining; however, the rate of chemical reaction and the intricate interplay between mechanical and chemical reactions during the process constitute complex dynamics in dynamic equilibrium. Further research is imperative to validate the formation and breaking of chemical bonds, facilitating a quantitative analysis of the impact of chemical reactions and unveiling the underlying mechanism of these reactions. This nuanced understanding would significantly enhance the predictability of the processing outcomes.

(5) To fulfill the need for the efficient attainment of ultra-smooth surfaces, composite polishing, grounded in the distinctive characteristics of diverse machining methods, emerges as a promising approach. For instance, the swift convergence of surface shape accuracy can be accomplished by employing PP. Subsequently, the remarkable efficiency of MRF is harnessed to diminish the surface roughness to the nanometer or sub-nanometer level while preserving the surface shape accuracy to the greatest extent possible. Ultimately, through iterative processes such as CMP, IBF, or EEM, further reductions in surface roughness, even reaching the sub-angstrom level, can be achieved to effectively meet the stringent requirements of significant scientific facilities. In addition to combination machining, the introduction of external energy can enhance the efficiency of contemporary industrial production processes to a certain extent. For instance, recent years have witnessed widespread attention towards ultrasound vibration-assisted polishing, plasma-assisted surface modification, and other electrochemical and photochemical auxiliary methods. The specific application combination also needs to be comprehensively considered in conjunction with the material characteristics, process requirements, and other factors.

## Figures and Tables

**Table 1:** Optical material properties of fused silica, monocrystalline silicon, silicon carbide, and sapphire.

Name	Component	New Mohs	Chemical Activity	Processing Difficulty
Fused silica	SiO_2_	7	Relatively stable under common chemical substances and resistant to acid and alkali	It is easy to crack during machining and integrity of crystal structure needs to be considered
Silicon	Si	7	Oxidation reaction occurs under the action of high temperature and oxygen	Cracks occur during machining, and there is often an oxide layer on the surface
Silicon carbide	SiC	13	Oxidation reaction occurs under the action of high temperature and oxygen	Material removal is difficult to achieve due to high hardness
Sapphire	Al_2_O_3_	12	Possible hydrolysis when encountering strong alkalis	High hardness prone to cracking during processing

**Table 2:** Comparison of technical performance of ultra-smooth surface processing.

Processing Method	Machining Accuracy	Processing Efficiency	Characteristics	Application Advantages	Limitations	Applications
Chemical mechanical polishing	Sub-nanometer/Sub-angstrom	√√√	Chemical reaction modified and softened.	Numerous materials that can be processed and achieve ultra-low surface roughness.	The removal efficiency is low and the stability is insufficient, mainly used for machining flat and low-curvature workpieces.	Optical components; integrated circuits.
Elastic emission machining	Sub-nanometer/Sub-angstrom	√	Hydrodynamic pressure produces shear stress.	High machining accuracy, atomic-level elimination, and no subsurface damage.	High precision requirements for equipment movement and low polishing efficiency.	Hubble Space Telescope; James Webb Space Telescope.
Magnetorheological finishing	Sub-nanometer	√√√	Magnetic fields regulate the viscosity of magnetorheological fluids.	High machining precision and numerous materials that can be processed.	Magnetorheological fluids have high costs, insufficient stability, and small scratches on the machining surface.	Hemispherical resonator of gyroscope; large astronomical telescopes.
Ion beam finishing	Sub-nanometer/Sub-angstrom	√√	Elastic collision, energy transfer, physical sputtering.	High machining precision, suitable for complex surface shapes, stable removal function, and no pollution.	The equipment is expensive, and the vacuum environment limits the processing size.	DUV; EUV; laser systems.
Plasma polishing	Nanometer/Sub-nanometer	√√√√	Active particles promote chemical reaction removal on the surfaces of materials.	Suitable for large-scale and complex surface polishing and no pollution.	The processing area can easily produce material oxidation and sputtering deposition.	Lightweight space optical systems.

## Data Availability

Not applicable.
